# Multi-criteria decision analysis approach for strategy scale-up with application to Chagas disease management in Bolivia

**DOI:** 10.1371/journal.pntd.0009249

**Published:** 2021-03-26

**Authors:** Maria-Jesus Pinazo, Ainize Cidoncha, Gurram Gopal, Silvia Moriana, Ruth Saravia, Faustino Torrico, Joaquim Gascon

**Affiliations:** 1 Barcelona Institute for Global Health (ISGlobal), Hospital Clínic—University of Barcelona, Barcelona, Spain; 2 Illinois Institute of Technology, Chicago, Illinois, United States of America; 3 Polytechnic University of Catalonia, Barcelona, Spain; 4 Chagas Disease Global Coalition, Barcelona, Spain; 5 Fundación CEADES, Cochabamba, Bolivia; University of Oxford, UNITED KINGDOM

## Abstract

**Objective:**

Design and build a strategy construction and evaluation software system to help stakeholders to develop viable strategies to expand (and adapt) the Chagas Platform healthcare model through the primary healthcare system in Bolivia.

**Methods:**

The software was built based on a ranking of medical Interventions and Actions (needed to support Interventions’ implementation) needed for comprehensive management of Chagas Disease in Bolivia. The ranking was performed using a Multi Criteria Decision Analysis (MCDA) methodology adapted to the WHO’s building blocks framework. Data regarding the criteria and the rankings was obtained through surveys and interviews with health care professionals working on Chagas disease. The Analytical Hierarchy Process was used to construct the decision criteria weights. Data Envelopment Analysis was used to identify the Interventions that lay on the efficiency frontier of outcomes and the complexity of associated Actions. These techniques were combined with integer programing tools using the open-source software R to build a decision-making tool to assess the outcomes and complexity of any combination of Interventions and Actions. This model and tool were applied to data concerning the care of Chagas disease in Bolivia collected through surveys of experts. The tool works by loading the data from each specific context.

**Results:**

The initial set of Interventions and Actions recommended after analysis of the survey data was further refined through face-to-face interviews with field experts in Bolivia, resulting in a strategy of 18 Interventions and 15 Actions. Within the WHO model the Leadership and Governance building block came up as the one needing more support with Actions such as the inclusion of Chagas into Annual Municipal Operational Plans by appointing local and provincial coordinators.

**Conclusion:**

This project established the suitability of the model for constructing healthcare strategies. The model could be developed further resulting in a decision-making tool for program managers in a wide range of healthcare related issues, including neglected and/ or prevalent diseases. The tool has the potential to be used at different stages of decision making by diverse stakeholders in order to coordinate activities needed to address a health problem.

## Author summary

This manuscript presents a strategy construction tool to generate viable strategies to expand the Chagas platforms for healthcare pilot through the primary healthcare system in Bolivia. Relevance of this work resides in the need for performing a rational planification to address Neglected Diseases in low- and middle-income countries, by prioritizing Interventions and Actions. After gathering and loading the data from each specific context and problem, policy makers can use the tool for construction or evaluation of strategies using the WHO Building Block model. The main comparative advantage is that the model does not only include the identification and evaluation of Interventions for the comprehensive care of Chagas, but also includes the necessary activities in the health system to support the large-scale implementation of them. The tool has the potential to be used at different stages of decision making to coordinate activities to address a health problem. The tool could be adapted for its use in other neglected and/ or prevalent diseases or in other locations.

## Introduction

Scaling up is defined by the WHO as efforts to increase the impact of innovations successfully tested in pilot or experimental projects, thus benefitting more people and fostering policy and program development on a lasting basis.[1] To achieve this aim it is important to plan and develop a good scaling up strategy.[1–3] Moreover, the resources in many cases are significantly limited and prioritizing them is extremely important. [4]

Most project decisions and program evaluations cannot be based on a single criterion as there are many different impact areas that need to be considered. MCDA enables the ranking of options depending on their impact on different areas and from the perspective of different stakeholders [5]. Previous studies [6–8] used MCDA strategy for prioritizing Interventions but did not take into account the supporting Actions that may be required to be able to perform them. In this project we adapted WHO’s building blocks model of healthcare systems evaluation and used MCDA strategy to prioritize two different types of investments, those related to the service provided to the patient (Interventions) and the those involving the managerial actions to enhance the actual health care system (Actions).

Chagas disease (CD) is a life-threatening illness caused by the *Trypanosome cruzi* parasite infecting 6 to 7 million people worldwide [9]. Its economic burden is similar to those of other prominent diseases globally (up to $7·19 billions of global costs per year)[10]. Latin America is the region most affected by Chagas disease, where it is one of the main public health issues. Although vector control activities have been implemented and two drugs are available for treatment of the disease less than 1% of the infected population are diagnosed and treated [11].

Bolivia has the highest prevalence of CD in the world (6.1%)[9]. Despite CD being a priority for the Bolivian Ministry of Health (MoH)[12], there is no regulation for implementing comprehensive care for people at risk of having *T*. *cruzi* infection. The situation of CD care in the country, despite the efforts of the National Chagas’ Program (PNCH), has still not been resolved, and serious situations of inaccessibility to quality, equitable and efficient health services that adequately respond to the needs of the affected groups persist. Some of the main causes of exclusion in health of populations at risk and Chagas patients are the structures of the National Health System (NHS), decentralized at Departmental Level that do not provide consistent and systematic diagnostic and treatment guidelines for Chagas patients. The norms, standards and tools available in the NHS for the comprehensive management of the patient between different levels of care are not sufficiently developed. Due to the scarcity of resources, in the 1990’s and early 2000’s, prevention strategies focused on vector control and mother-to-child transmission. By 2009, there was no comprehensive strategy for management of *T*. *cruzi* infection in adults.[3]

ISGlobal and CEADES have run a pilot in 6 centers in Bolivia during the past 6 years treating Chagas patients. This research focuses on building, from the experience of the Chagas Platforms a tool to help find the best way of integrating the care of Chagas disease into the national primary health care system.[3]

## Methods

### Objective of the work

The main objective of the study was to build a software-based strategy construction tool to help stakeholders to generate viable strategies to expand the Chagas Platform healthcare model through the primary healthcare system in Bolivia.

### Framework

We adapted the widely used WHO’s building blocks model [12] as a framework to evaluate the outcomes and necessities of a Health Care system. The aim was to develop a computational model based on this framework to prioritize the integral care Interventions to be provided and the supporting Actions needed to enhance each of the Health Care system’s building blocks. The WHO’s framework identified four main outcomes criteria (improved health -level and equity-, responsiveness, financial risk protection and improved efficiency), and six building blocks (leadership/governance, health care financing, health workforce, medical products and technologies, information and research, and service delivery). Since these outcomes criteria and building blocks are too broad for evaluation, deeper and more specific sub-criteria are needed for an effective MCDA evaluation [13].

**Fig 1:**
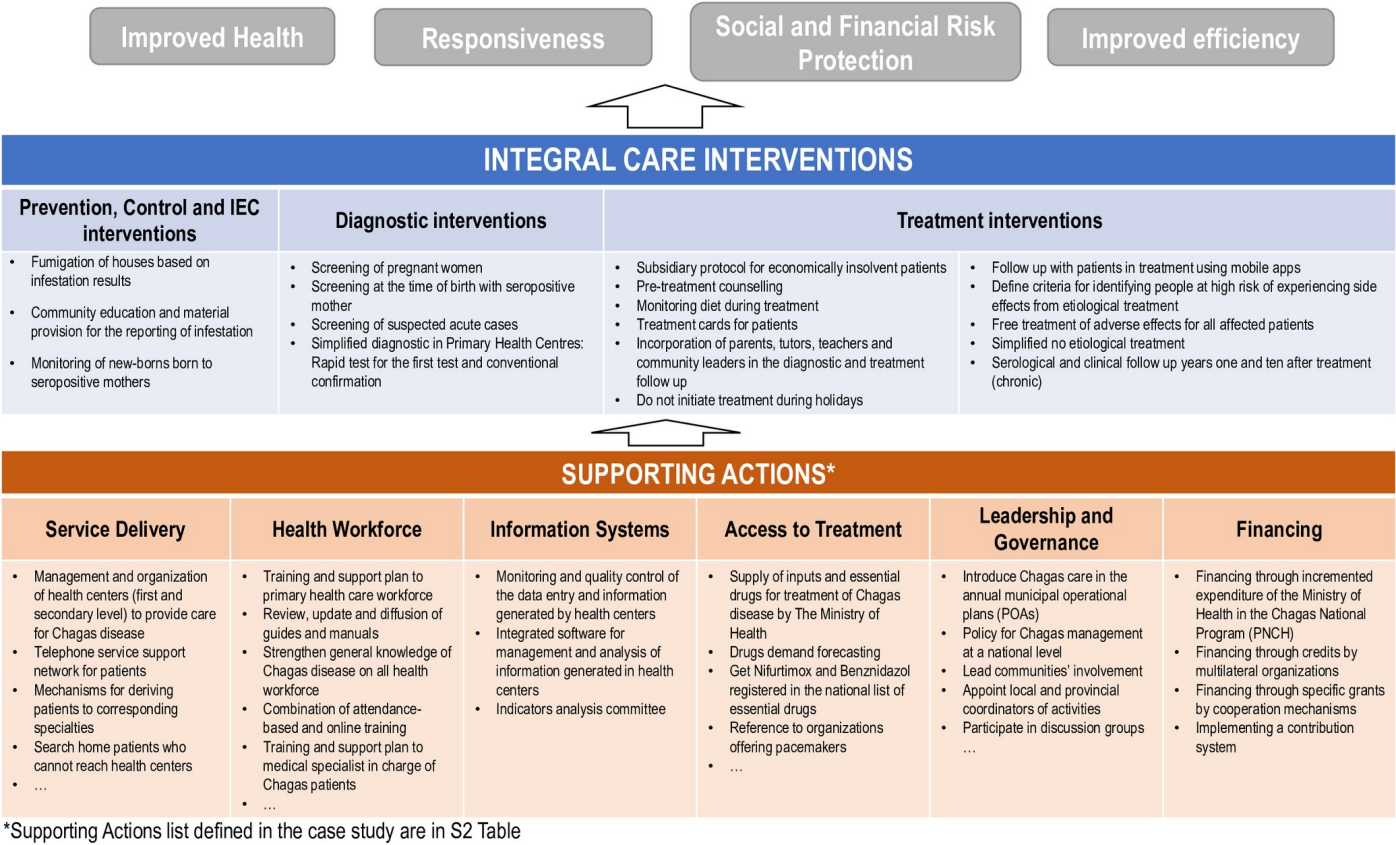
Model to evaluate Interventions and Actions.

The identification, characterization and grouping of the options (Interventions and Actions) are crucial for choosing an appropriate way of modeling so that scaling-up strategies could be determined. The possible options to be implemented came from a variety of sources including experiences and programs already implemented in other countries. [14–16] We generated a list of nearly 200 options and reviewed all of them. We narrowed the list by grouping those similar options and by deleting redundant ones. The options fell into two main different groups. One group related to the care provided to the patient directly impacting the disease management in terms of control, prevention, education, diagnostic and treatment; we call these “**Interventions**” (see the list of Interventions in the S1 Table). The other group was composed of supporting activities needed in the Primary Health Care system in order to be able to perform the Interventions; we call these “**Actions**” (see the list of Actions in the S2 Table). This resulted in a complex model (Fig 1) that could let us evaluate the two main groups of options (Interventions and Actions) and the relationships between them.

### Adaptation and combination of standard mathematical methods

We used R (version 2.11.1) and R studio (version 1.0) to develop a Multi Criteria Decision Analysis model, combining and adapting the standard AHP[17], MDCA[18] and DEA[19] models to our problem structure.

In a standard MDCA model there are two main elements: the options or Interventions to be evaluated and the multiple criteria used for that aim. In our model there is an extra element, the Actions supporting the health system’s building blocks.

Each Intervention I_j_ results in outcomes in the four outcome categories; however, each Intervention also has associated with it a complexity metric for each of the six building blocks. The complexity metric measures the difficulty in the scale-up of the Intervention to produce the expected outcomes. Our model adds to the literature in that we identify a set of Actions that can be taken to reduce the complexity in each Building Block for the set of chosen Interventions. In order to accomplish this, we included a new level in the complexity side of our performance matrix adding all the possible Actions required by the selected Interventions to address the complexity of the Interventions and improve their scale-up effectiveness. Therefore, we needed to compute another level of weights for each possible Action.

First, we used AHP in order to compute the relative weights for the decision criteria of the model using a pairwise comparison matrix obtained through surveys of a select group of decision makers (S1 Text). Relative weights for the “complexity” criteria identified in the six “Building Blocks” set of the model were determined in the same survey evaluating their complexity on a scale from one to four.

Once the criteria weights were established, each potential “Intervention” was evaluated by its impact on the criteria in the “outcomes” group, and necessity of “Actions” on each of the “Building Blocks”. It is noted that the Building Blocks are needed to enable the outcomes associated with an Intervention- if more Actions from the building blocks are needed this increases the “complexity” associated with the success of an Intervention. For each supporting “Action” we determined its contribution to addressing the complexity of every Building Block criterion. The maximum potential for addressing each building block criterion’s complexity was determined by summing up the contributions of all the Actions supporting that criterion. Dividing the actual contribution towards a criterion by the maximum potential for that criterion yielded a relative effectiveness factor for each of the Actions. For instance, if A_1_,…, A_r_ are Actions related to the criteria C_1_ the contribution potential or weight for Action Aj, represented as w_Aj_ is computed as follows:

$$w_{A_{j}}=\frac{A_{j}}{A_{1}+A_{2}+\cdots +A_{n}}*w_{C_{1}}$$

Once we have one specific weight for each of the columns of the performance matrix the computation of the Outcomes and Complexity scores is automatically performed by the tool, through the standard MCDA performance matrix. (S2 Text)

### Finding optimal combinations by iterative construction

Although individual scores can give us an idea of the preferred Interventions our objective was to determine the most suitable combinations of Interventions and Actions for implementation. To do this we define the outcomes of a combination as the sum of the outcomes mark of each selected Intervention. It should be noted that Actions are one-time implementations that can support more than one Intervention, hence they are counted only once towards their effect on the Building Block. For instance, if Interventions I1 and I2 are selected and both require Action A3 we only need to add the weight of this Action once towards the building block criteria. To simplify our analysis, we make the following assumptions:

- The Interventions do not overlap and are additive. Whenever an Intervention is added to the strategy its outcomes are added to the overall outcomes of the strategy.
- All the Actions except the financial ones are one-time Actions, which means that if the Action is required for more than one Intervention in the Strategy its potential is added only once.
- The estimated unitary cost, the estimated impact in the public budget and the Actions for the financing party are assumed to be independent for each Intervention.

**Fig 2:**
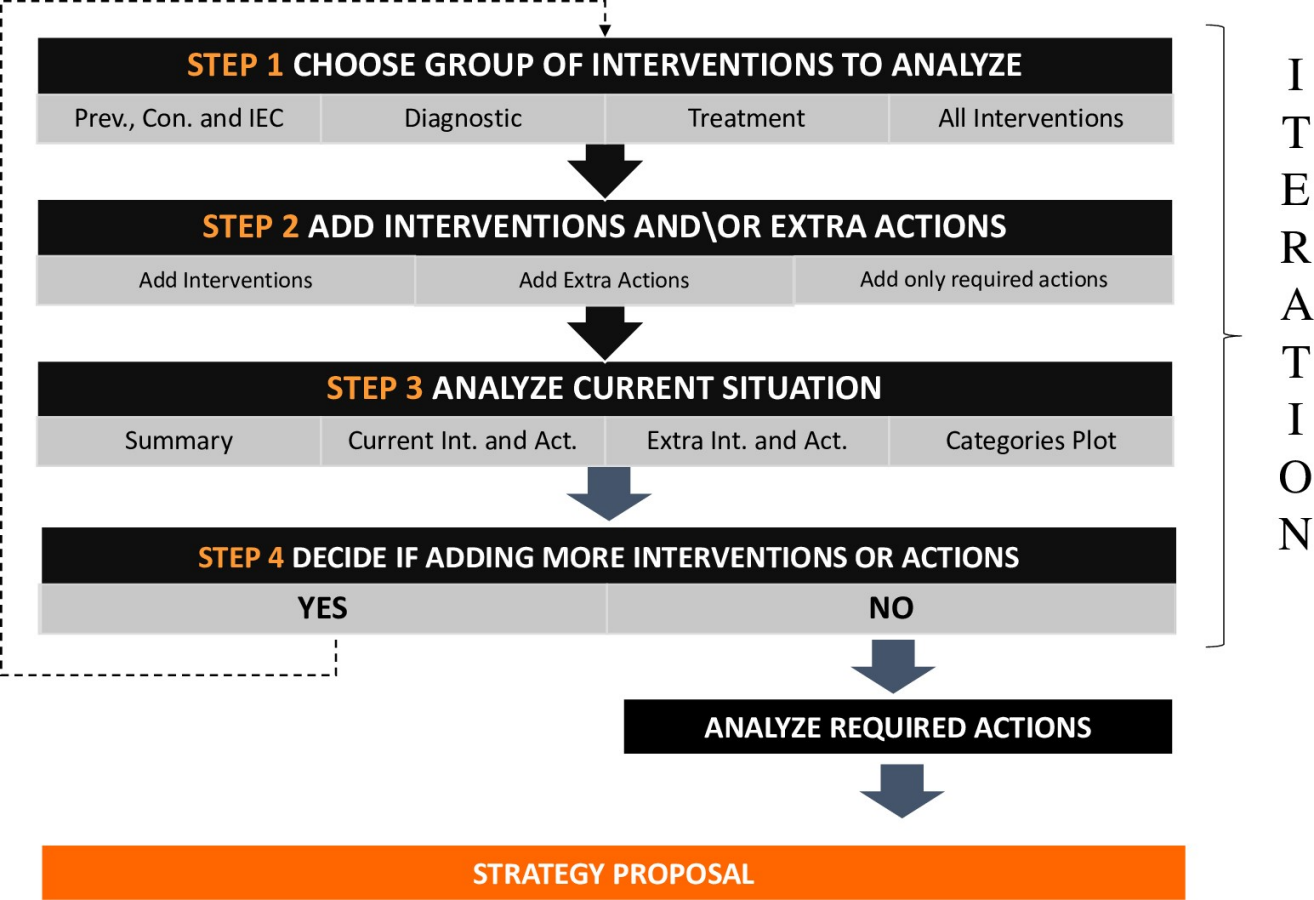
Iterative process step by step to select Actions and Interventions.

Based on these assumptions we developed a tool in R in order to allow us to iteratively construct a strategy with the experts and help them in their decision-making process. The steps of the process, illustrated in Fig 2, are the following:

- **STEP 1: Choose a group of Interventions to analyze.** The algorithm starts by allowing the user to choose which type of Interventions she/he want to focus on, or if she/he want to analyze all Interventions at once. The tool will then show the analysis of outcomes and complexity for each selected Intervention.
- **STEP 2: Add Interventions and\or extra Actions.** The user will now be able see a graph with the Interventions plotted by Outcomes and Complexity as well as a table to sort and select the Interventions that the user wants to add on this iteration. Once the users have analyzed the possibilities he/she is able select the most suitable Interventions and/or Actions to add to their strategy.
- **STEP 3: Analyze the current situation.** The tool will show a summary of the updated Strategy, with the Interventions and Actions on them together with the achieved Outcomes and Complexity.
- **STEP 4: Finalize or revise strategy.** At this point the user is able to decide whether they want to finalize the strategy as it is or if they want to start a new iteration.

If no more Iterations are needed: The user is able to analyze in more detail which are the Actions required for the Interventions on their strategy and build a Strategy proposal.

If more Iterations are needed: The tool would go back to Step 1 for the user to keep adding Interventions.

### Data for Bolivia case study

In the specific context of the study, Interventions’ and Action’s lists were built based on a selection of Interventions and Actions defined in Bolivia and in other Latin American countries with Chagas Disease specific programs. Following the mentioned review, some new approaches that could be interesting to evaluate were added to Bolivia’s Chagas National Program strategy approaches. As a result of this exercise, we generated a list of nearly 200 Interventions and reviewed them with subject matter experts from ISGlobal, CEADES and the Chagas Global Coalition. We narrowed the list by grouping some Interventions and deleting redundant ones and further clarified some Interventions. An intensive collaborative work was done in this area since the different groupings and categorization of the strategies was crucial for the design of the model. The specific criteria to be used within the Outcomes and the Building Blocks sections were also determined through a review of literature and consultations with subject matter experts.

Once the criteria and the Interventions were determined we performed a number of different surveys in order to obtain expert knowledge to feed our model.

The outcome and complexity criteria weights were based on a pairwise comparison survey to which AHP methodology was applied. In the same survey we included a list of possible Actions that could support the building blocks and we asked the respondents to rank their complexity on a four points scale. Following the process explained in the methodology section we first determined the importance of each criteria by allocating weights and then we computed the individual weights for each of the Actions based on their complexity and the weight of the criteria they are supporting.

The performance matrix for Interventions was based on surveys completed by experts in each area- thus different surveys were carried out depending on the area of expertise needed for the set of Interventions. These experts were asked to choose the outcomes a specific Intervention was impacting and the supporting Actions that were required to make that Intervention feasible in the current situation.

### Ethics

Current study does not involve individuals in terms of intervention (no patients), given that the main aim was the development of a decision-making model, based on general information about Chagas Disease and how Health System may address it in a specific setting. Surveys and interviews performed with experts were voluntary, anonymized, and the responses were not recorded, but registered in documents only available for the research team. Before being involved in the study, enough time was devoted to explain to the participants the aim, purpose, methodology, expected results and its potential use in building the software., as well as to answer all their questions and concerns.

## Results

### Interventions—individual ranking

**Fig 3:**
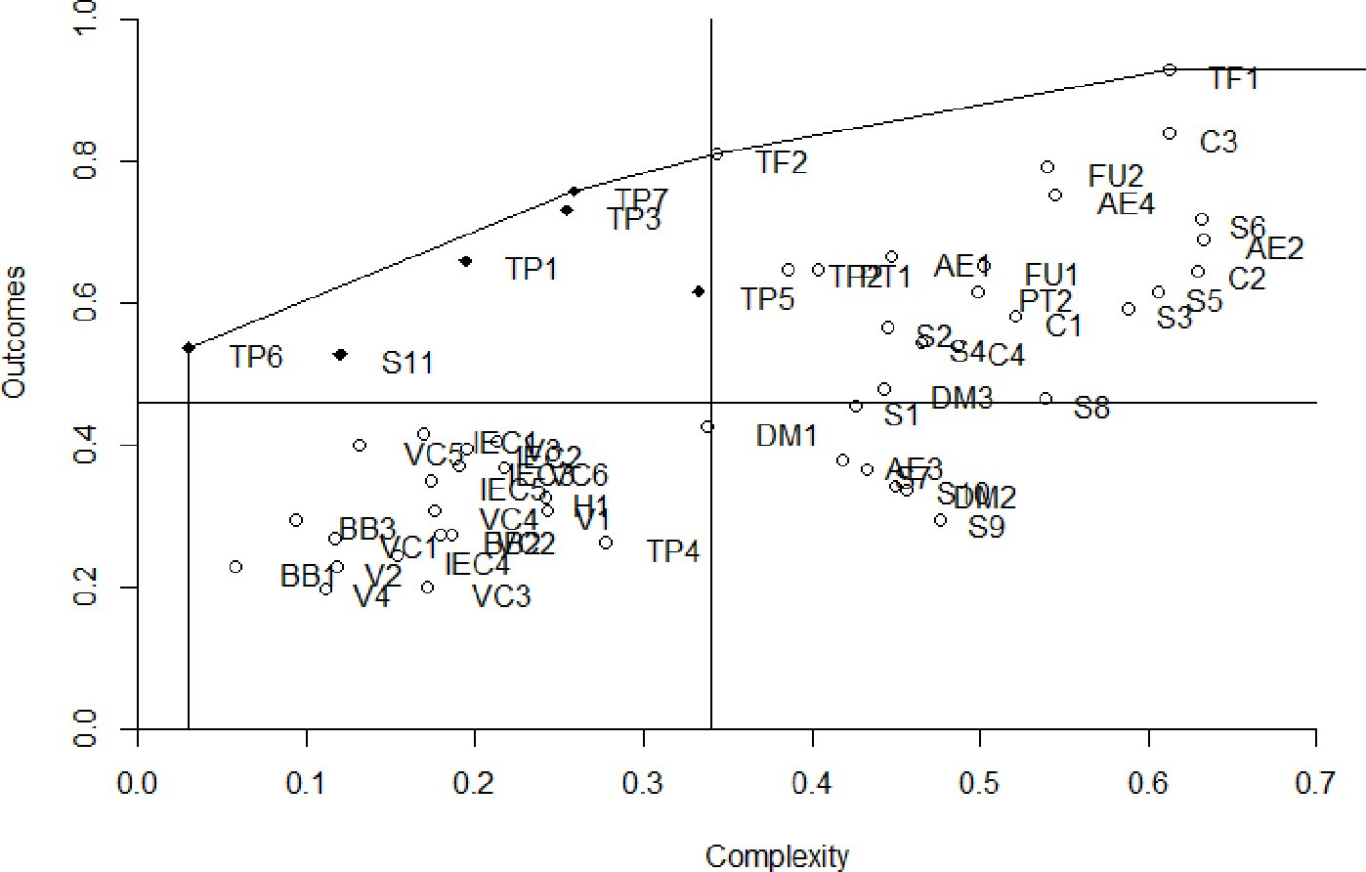
Selected Interventions’ complexity and outcomes representation.

An individual ranking of each of the Interventions was developed based on their Complexity and Outcomes score (Fig 3). The median lines are drawn in the figure in order to divide the Interventions into four categories.

In the first category, which contains the Interventions with high outcomes and low building blocks complexity we find Interventions related to the process of etiological treatment, as *TP3-Incorporation of parents*, *tutors*, *teachers and community leaders in the diagnostic and treatment*, which can be implemented once we have enough tools to provide the treatment. The S*11- Screening of acute cases* is also a highly recommended Intervention and is noteworthy as stands out from other screening Interventions.

Patients’ financially related issues (*TF1* and *TF2*) have high outcomes marks. In addition, *TF2- Subsidiary protocol for economically insolvent patients*, even though it does not fit in Category 1 has a very high rank in Outcomes and it implies almost half of the complexity of *TF1- Free diagnostic and treatment for all patients* so it might be an Intervention to be considered for implementation.

### Actions—individual ranking

From the aggregated performance matrix, we can compute the number of Interventions supported by each Action and also the Outcomes that they indirectly imply. These outcomes are the sum of the outcomes scores of each of the Interventions for which the Action was selected.

In the set of Actions by building block (Table 1) most of the chosen Actions supported than half of the Interventions except the ones in Italics. These Actions were selected due to their low assessed complexity and the high number of Interventions they support (>40%). *BHW13-Combination of presently and online training* was selected even though it supported only 39% of the Interventions as the other two related options *BHW11-Entirely presently training* and *BHW12-Entirely online training* supported only 6% and 0% of the Interventions respectively.

**Table 1:** Actions defined classified by building block.

Most required Actions					
Service delivery (SD)	Health workforce (HW)	Information System (I)	Access to medicine (MVT)	Financing (F)	Leadership and governance (LG)
-Management and organization of health centers (first and secondary level) to provide care for Chagas disease	-Strengthen general knowledge of Chagas disease on all health workforce -Training and support plan to primary health care workforce -Combination of attendance-based and online training -Review, update and diffusion of guides and manuals	-Monitoring and quality control of the data entry and information generated by health centers	-Supply of inputs and essential drugs for treatment of Chagas disease by The Ministry of Health	-Financing through incremented expenditure of the Ministry of Health in the Chagas National Program (PNCH)	-Introduce Chagas care in the annual municipal operational plans (POAs) -Policy for Chagas management at a national level -Lead communities’ involvement -Develop an intervention plan

### Combinations

Using an iterative tool developed in R, which computes the combined complexity as explained in the methodology we got the following First strategy proposal (S3 Table).

## Discussion

### From results

After conducting the surveys and our data analysis and having developed the decision-making model we traveled to different areas of Bolivia including the city of Cochabamba and the rural area of Punata. During this visit we met with various stakeholders who have been classified as Chagas Platforms Health Workforce and Bolivia’s public health managers. We identified the main areas where the stakeholders agreed with the model results and noted areas of disagreement. Table 2 summarizes the main discussion points during our visit.

**Table 2:** Main discussion results with local stakeholders.

	CONFIRM	REJECT
**AGREE**	INTERVENTIONS: • Prevention, control and information, education and communication (IEC) already integrated • VC5- Community education and material provision to report infestation • S2-Screening pregnant women • S4- Screening new-born from positive mother • S11- Screening of acute cases • C3- Simplified non-etiological treatment ACTIONS: Emphasis on BSD13, BW14, BI3, BMVT1 and BLG2	INTERVENTIONS: • TP1- Following up diet • TP6- Not to initiate treatment on holidays periods • Diagnostic methods context-sensitive
**DISAGREE**	• AE4- Free treatment on adverse effects to all affected patients • Diagnostic methods	

Based on this discussion the Interventions *TP1-Following up the diet* and *TP6-No entry to treatment on holidays* were removed from the strategy. Despite the discussion we decided to keep *AE4- Free treatment of adverse effects for all affected patients* since it seems to be an effective Intervention if the terms are agreed. Regarding the Diagnostics methods, although there was no clear agreement, *DM1-Diagnostic using the two conventional methods* seemed eventually more appropriate for the current situation so it replaced *DM3-Simplified diagnostic on Primary Health Care* on our previous strategy proposal.

### Sensitivity analysis

In order to assess the sensitivity of our results we collected two more criteria-related surveys in Bolivia and incorporated them into the analysis. We could thus assess the changes to the model and the sensitivity of our results to the variation in criteria weights. The result was a maximum difference of 0.07 on outcomes and 0.03 on complexity.

There are two main changes in the results. There is a change on outcomes ranking between *DM1-Diagnostic using the two conventional methods* and *S8-Screening family of positive cases*.—*DM1* now lies in Category one and *S8* drops to Category four. On the other hand, the change in complexity makes *TF2- Subsidiary protocol for economically insolvent patients* lie as well in Category one and drop *TP5- Assure suspension of treatment when indicated* to Category two. This would result in the inclusion of *DM1* and the drop of *TP*5 from the top Interventions. However, *TP5* is one of the Interventions that we assessed as necessary due to security. It is not an option and will be always included in our Interventions. Referring to *DM1-Diagnostic using 2 conventional methods*, this change confirms one of the conclusions from the discussion with experts in Bolivia and the highlights the current situation of not having a *DM3-Simplified diagnostic on Primary Health Care* option.

### Final proposal

Starting with our First Proposal and considering the above-mentioned discussions we iteratively constructed a new strategy leading to the final proposal, shown in Table 3.

**Table 3:** Final Strategy proposed to control Chagas disease in Cochabamba, Bolivia.

**Part 1 of the strategy: Health Interventions to control Chagas disease**		
**Code**	**Description**	
**Prevention, Control and IEC**		
BB1	Screening people donating blood banks	
V3	Monitoring of new-borns born to seropositive mothers	
VC1	Fumigation of houses based on infestation results	
VC5	Community education and material provision for the reporting of infestation	
**Screening and diagnosis**		
S2	Screening of pregnant women	
S4	Screening at the time of birth with seropositive mother	
S11	Screening of suspected acute cases	
DM1	Diagnostic using 2 conventional methods	
**Treatment and follow up**		
TF2	Subsidiary protocol for economically insolvent patients (for treatment)	
PT1	Pre-treatment counselling	
TP2	Treatment cards for patients follow up	
TP3	Incorporation of parents, tutors, teachers and community leaders in the diagnostic and treatment	
TP5	Assure suspension of treatment when indicated	
TP6	Do not initiate treatment during holidays	
TP7	Follow up with patients in treatment using mobile apps, e.g. Whatsapp	
AE1	Define criteria for identifying people at high risk of experiencing side effects from etiological treatment	
AE4	Free treatment of adverse effects for all affected patients	
C3	Simplified no etiological treatment(provided by doctors in provincial hospitals and not by specialists in advanced hospitals)	
FU2	Serological and clinical follow up years one and ten after treatment (chronic)	
**Part 2 of the strategy: Actions to strengthen health system to support Interventions**		
***Code***	***Description***	***Number of Interventions supported***
BLG2	Introduce Chagas care in the annual municipal operational plans (POAs)	13
BSD13	Management and organization of health centers (first and secondary level) to provide care for Chagas disease	12
BHW4	Training and support plan to primary health care workforce	12
BHW14	Review, update and diffusion of guides and manuals	12
BHW2	Strengthen general knowledge of Chagas disease on all health workforce	11
BMVT3	Supply of inputs and essential drugs for treatment of Chagas disease by The Ministry of Health	11
BLG9	Policy for Chagas management at a national level	11
F1	Financing through incremented expenditure of the Ministry of Health in the Chagas National Program (PNCH)	11
BI1	Monitor and quality control of the data entry and information generated by health centers	10
BHW13	Combination of attendance-based and online training	9
BI3	Integrated software for management and analysis of the information generated in the health centers	9
BLG10	Appoint local and provincial coordinators of the activities	9
BMVT1	Drugs demand forecasting	8
BLG5	Lead communities involvement	8
BLG12	Participate in discussion groups for technical support in developing / updating the national protocol for diagnosis and treatment	8
BSD3	Telephone service support network for patients	7
BLG1	Develop an intervention plan	7
BHW8	Training community volunteers and vector control staff on entomological surveillance	6
BHW17	Accountability of attended cases by professionals (for later analysis of congruence with prevalence in the area)	6
BI2	Indicators analysis committee	6
BLG14	Joint action agreements with institutions and health services in health network	6
BSD2	Mechanisms for deriving patients to corresponding specialties (promoting inter-institutional agreements)	5
BSD7	Search home patients who cannot reach health centres	5
BHW3	Training and supporting plan to specialist which are in charge of Chagas patients	5
BMVT2	External support in supply chain from the national program (stocks-out supporting networks, circuit shopping. . .)	5
BLG6	Meetings and advocacy workshops in front of representatives from institutions and organizations	5
BLG8	Leadership and program support of political representatives	5
BSD6	Health professionals outreach activities (going to houses and rural areas)	4
BSD10	Enhancement of laboratories	4
BHW9	Assist and monitor all diagnostic and treatment activities in the field	4
BHW15	More hours dedicated to treatment and diagnosis of Chagas in the medical curriculum	4
BLG11	Improve the inter-institutional communication by meeting for consensus and agreements	4
F3	Financing through credits by multilateral organizations	4
BHW5	Training and supporting HW from relevant related programs	3
BHW7	Training possible in charge of peers previously trained	3
BMVT5	Get Nifurtimox and Benznidazol registered in the national list of essential drugs	3
BSD1	Mechanisms and flowcharts of care for people detected in prevention and control activities	2
BSD9	Mobile cardiograms	2
BSD11	Equipment improvement	2
BHW1	Additional Human Resources	2
BHW6	Training in charge of MOH professionals and departmental Chagas representatives.	2
BHW10	On the job training (internships. . .)	2
BLG3	Assistance to scientific events, local, regional and international	2
BLG7	Publication of articles, manuals and other informative materials for expanding and socializing the model	2
BLG13	Activities for validation of a new simplified diagnostic protocol	2
BLG15	Chagas law	2
BSD5	Performing regular external quality control on laboratories	1
BMVT4	Reference to organizations offering pacemaker	1
F2	Financing through specific grants by cooperation mechanisms	1
F4	Implementing a contribution system	0

### The model and its limitations

The model used for this project can lead to a new process for analyzing and constructing strategies for different diseases or in different countries.

There are several areas for improving and refining the study. Data definition and collection is crucial for this project. Interventions should be defined clearly in order to avoid misunderstandings. In some cases, it is hard to describe an Intervention or Action within a line but it is important to ensure a common interpretation. The design of the surveys also can be improved. It is a challenge to collect the detailed data that is required and finding a way to make it easier and less time consuming for the experts would facilitate the data collection process. Conducting the surveys in the local language is a necessity, and thus translation and back translation of surveys is essential for the data to be meaningful.

One of the most highlighted points during the discussion with the experts in Bolivia was the importance of the local context. Even though survey respondents were asked to take the current Bolivia’s situation into account, there were notable differences within the local context in the country. Three main scenarios were mentioned: Urban, Peri-Urban and Rural. However, it is possible that even with this classification there are still high variations by department or even by municipality.

## Conclusions

This model has proven useful for policy-makers who have to design strategies for scaling up health innovations considering multiple variables and criteria. A wide range of expertise is needed in the different steps of the study. To ensure the quality of results it is critical to have the right number of participants with representation of different domains, from experts to health professionals to patients, and with proper understanding of the contexts and subject of study. The tool supports the selection of Interventions based on impact and complexity criteria, but the development of a final coherent strategy and the proposal relies on a process of consensus among the strategy owners.

Finally, the model can be adapted for use in different domains and situations, and it can assist managers in making more analytical and informed decisions.

## Supporting information

S1 Table List of Interventions. (DOC)

S2 Table List of Actions. (DOC)

S3 Table First strategy proposal defined with the use of the tool. (DOC)

S1 Text Survey to experts in order to establish relative weight of each intervention. (DOCX)

S2 Text Mathematical details to build the model. (DOCX)
