# Supplementary material for: Multi-criteria decision analysis approach for strategy scale-up with application to Chagas disease management in Bolivia
Source: PLoS Negl Trop Dis. 2021 Mar 26;15(3):e0009249. doi: 10.1371/journal.pntd.0009249 (PMC8026069; doi:10.1371/journal.pntd.0009249)
Supplement: S1 Table — (DOC) [file pntd.0009249.s001.doc]

**S1_Table_List of Interventions**

| **Health interventions to control Chagas disease** | | | |
| --- | --- | --- | --- |
| **Code** | | **Description** | |
| **Prevention, Control and IEC** | | | |
| VC1 | Fumigation of houses based on infestation results | |  |
| VC5 | Community education and material provision for the reporting of infestation | |  |
| V3 | Monitoring of new-borns born to seropositive mothers | |  |
| **Screening and diagnosis** | | |  |
| S2 | | Screening of pregnant women |  |
| S4 | | Screening at the time of birth with seropositive mother |  |
| S11 | | Screening of suspected acute cases |  |
| DM3 | | Simplified diagnostic in Primary Health Centres: Rapid test for the first test and conventional confirmation |  |
| **Treatment and follow up** | | |  |
| TF2 | | Subsidiary protocol for economically insolvent patients (for treatment) |  |
| PT1 | | Pre-treatment counselling |  |
| TP1 | | Monitoring diet during treatment |  |
| TP2 | | Treatment cards for patients follow up |  |
| TP3 | | Incorporation of parents, tutors, teachers and community leaders in the diagnostic and treatment |  |
| TP5 | | Assure suspension of treatment when indicated |  |
| TP6 | | Do not initiate treatment during holidays |  |
| TP7 | | Follow up with patients in treatment using mobile apps, e.g. Whatsapp |  |
| AE1 | | Define criteria for identifying people at high risk of experiencing side effects from etiological treatment |  |
| AE4 | | Free treatment of adverse effects for all affected patients |  |
| C3 | | Simplified no etiological treatment(provided by doctors in provincial hospitals and not by specialists in advanced hospitals) |  |
| FU2 | | Serological and clinical follow up years one and ten after treatment (chronic) |  |
