# Supplementary material for: Multi-criteria decision analysis approach for strategy scale-up with application to Chagas disease management in Bolivia
Source: PLoS Negl Trop Dis. 2021 Mar 26;15(3):e0009249. doi: 10.1371/journal.pntd.0009249 (PMC8026069; doi:10.1371/journal.pntd.0009249)
Supplement: S2 Table — (DOC) [file pntd.0009249.s002.doc]

**S2_Table_List of Actions**

| **Actions to strengthen health system to support interventions** | | |
| --- | --- | --- |
| ***Code*** | ***Description*** | ***Number of interventions supported*** |
| BHW4 | Training and support plan to primary health care workforce | 14 |
| BHW14 | Review, update and diffusion of guides and manuals | 14 |
| BLG2 | Introduce Chagas care in the annual municipal operational plans (POAs) | 14 |
| BHW2 | Strengthen general knowledge of Chagas disease on all health workforce | 13 |
| BSD13 | Management and organization of health centers (first and secondary level) to provide care for Chagas disease | 12 |
| BHW13 | Combination of attendance-based and online training | 11 |
| BI1 | Monitoring and quality control of the data entry and information generated by health centers | 11 |
| BMVT3 | Supply of inputs and essential drugs for treatment of Chagas disease by The Ministry of Health | 11 |
| BLG9 | Policy for Chagas management at a national level | 11 |
| F1 | Financing through incremented expenditure of the Ministry of Health in the Chagas National Program (PNCH) | 11 |
| BI3 | Integrated software for management and analysis of information generated in health centers | 10 |
| BLG5 | Lead communities’ involvement | 10 |
| BMVT1 | Drugs demand forecasting | 9 |
| BLG10 | Appoint local and provincial coordinators of activities | 9 |
| BLG12 | Participate in discussion groups for technical support in developing / updating the national protocol for diagnosis and treatment | 9 |
| BLG1 | Develop an intervention plan | 8 |
| BSD3 | Telephone service support network for patients | 7 |
| BHW3 | Training and support plan to medical specialist in charge of Chagas patients | 6 |
| BHW8 | Training community volunteers and vector control staff on entomological surveillance | 6 |
| BHW17 | Accountability of attended cases by professionals (for later analysis of congruence with prevalence in the area) | 6 |
| BI2 | Indicators analysis committee | 6 |
| BMVT2 | External support in supply chain from national program (stocks-out supporting networks, circuit shopping...) | 6 |
| BLG14 | Joint action agreements with institutions and health services in the health network | 6 |
| BSD2 | Mechanisms for deriving patients to corresponding specialties ( promoting inter-institutional agreements) | 5 |
| BSD7 | Search home patients who cannot reach health centers | 5 |
| BHW15 | More hours dedicated to treatment and diagnosis of Chagas in the medical curriculum | 5 |
| BLG6 | Meetings and advocacy workshops in front of representatives from institutions and organizations | 5 |
| BLG8 | Leadership and program support of political representatives | 5 |
| BLG11 | Improve the inter-institutional communication by meeting for consensus and agreements | 5 |
| BSD6 | Health professionals outreach activities ( going to houses and rural areas) | 4 |
| BSD10 | Enhancement of laboratories | 4 |
| BHW9 | Assist and monitor all diagnostic and treatment activities in the field | 4 |
| F3 | Financing through credits by multilateral organizations | 4 |
| BSD1 | Mechanisms and flowcharts of care for people detected in prevention and control activities | 3 |
| BSD11 | Equipment improvement | 3 |
| BHW5 | Training and supporting health workforce from relevant related programs | 3 |
| BHW6 | Training in charge of Ministry of Health professionals and departmental Chagas representatives | 3 |
| BHW7 | Training in charge of peers previously trained | 3 |
| BMVT5 | Get Nifurtimox and Benznidazol registered in the national list of essential drugs | 3 |
| BLG15 | Chagas law | 3 |
| BSD9 | Mobile cardiograms | 2 |
| BHW1 | Additional Human Resources | 2 |
| BHW10 | On the job training (internships...) | 2 |
| BLG3 | Assistance to scientific events, local, regional and international | 2 |
| BLG7 | Publication of articles, manuals and other informative materials for expanding and socializing the model | 2 |
| BLG13 | Activities for the validation of a new simplified diagnostic protocol | 2 |
| BSD5 | Performing regular external quality control on laboratories | 1 |
| BMVT4 | Reference to organizations offering pacemakers | 1 |
| F2 | Financing through specific grants by cooperation mechanisms | 1 |
| F4 | Implementing a contribution system | 0 |
