# Supplementary material for: Multi-criteria decision analysis approach for strategy scale-up with application to Chagas disease management in Bolivia
Source: PLoS Negl Trop Dis. 2021 Mar 26;15(3):e0009249. doi: 10.1371/journal.pntd.0009249 (PMC8026069; doi:10.1371/journal.pntd.0009249)
Supplement: S3 Table — (DOC) [file pntd.0009249.s003.doc]

**S3_Table_First strategy proposal defined with the use of the tool**

| **Part 1 of the strategy: Health interventions to control Chagas disease** | | | |
| --- | --- | --- | --- |
| **Code** | | **Description** | |
| **Prevention, Control and IEC** | | | |
| VC1 | Fumigation of houses based on infestation results | |  |
| VC5 | Community education and material provision for the reporting of infestation | |  |
| V3 | Monitoring of new-borns born to seropositive mothers | |  |
| **Screening and diagnosis** | | |  |
| S2 | | Screening of pregnant women |  |
| S4 | | Screening at the time of birth with seropositive mother |  |
| S11 | | Screening of suspected acute cases |  |
| DM3 | | Simplified diagnostic in Primary Health Centres: Rapid test for the first test and conventional confirmation |  |
| **Treatment and follow up** | | |  |
| TF2 | | Subsidiary protocol for economically insolvent patients (for treatment) |  |
| PT1 | | Pre-treatment counselling |  |
| TP1 | | Monitoring diet during treatment |  |
| TP2 | | Treatment cards for patients follow up |  |
| TP3 | | Incorporation of parents, tutors, teachers and community leaders in the diagnostic and treatment |  |
| TP5 | | Assure suspension of treatment when indicated |  |
| TP6 | | Do not initiate treatment during holidays |  |
| TP7 | | Follow up with patients in treatment using mobile apps, e.g. Whatsapp |  |
| AE1 | | Define criteria for identifying people at high risk of experiencing side effects from etiological treatment |  |
| AE4 | | Free treatment of adverse effects for all affected patients |  |
| C3 | | Simplified no etiological treatment(provided by doctors in provincial hospitals and not by specialists in advanced hospitals) |  |
| FU2 | | Serological and clinical follow up years one and ten after treatment (chronic) |  |
| **Part 2 of the strategy: Actions to strengthen health system to support interventions** | | | |
| ***Code*** | | ***Description*** | ***Number of interventions supported*** |
| BHW4 | | Training and support plan to primary health care workforce | 14 |
| BHW14 | | Review, update and diffusion of guides and manuals | 14 |
| BLG2 | | Introduce Chagas care in the annual municipal operational plans (POAs) | 14 |
| BHW2 | | Strengthen general knowledge of Chagas disease on all health workforce | 13 |
| BSD13 | | Management and organization of health centers (first and secondary level) to provide care for Chagas disease | 12 |
| BHW13 | | Combination of attendance-based and online training | 11 |
| BI1 | | Monitoring and quality control of the data entry and information generated by health centers | 11 |
| BMVT3 | | Supply of inputs and essential drugs for treatment of Chagas disease by The Ministry of Health | 11 |
| BLG9 | | Policy for Chagas management at a national level | 11 |
| F1 | | Financing through incremented expenditure of the Ministry of Health in the Chagas National Program (PNCH) | 11 |
| BI3 | | Integrated software for management and analysis of information generated in health centers | 10 |
| BLG5 | | Lead communities’ involvement | 10 |
| BMVT1 | | Drugs demand forecasting | 9 |
| BLG10 | | Appoint local and provincial coordinators of activities | 9 |
| BLG12 | | Participate in discussion groups for technical support in developing / updating the national protocol for diagnosis and treatment | 9 |
| BLG1 | | Develop an intervention plan | 8 |
| BSD3 | | Telephone service support network for patients | 7 |
| BHW3 | | Training and support plan to medical specialist in charge of Chagas patients | 6 |
| BHW8 | | Training community volunteers and vector control staff on entomological surveillance | 6 |
| BHW17 | | Accountability of attended cases by professionals (for later analysis of congruence with prevalence in the area) | 6 |
| BI2 | | Indicators analysis committee | 6 |
| BMVT2 | | External support in supply chain from national program (stocks-out supporting networks, circuit shopping...) | 6 |
| BLG14 | | Joint action agreements with institutions and health services in the health network | 6 |
| BSD2 | | Mechanisms for deriving patients to corresponding specialties ( promoting inter-institutional agreements) | 5 |
| BSD7 | | Search home patients who cannot reach health centers | 5 |
| BHW15 | | More hours dedicated to treatment and diagnosis of Chagas in the medical curriculum | 5 |
| BLG6 | | Meetings and advocacy workshops in front of representatives from institutions and organizations | 5 |
| BLG8 | | Leadership and program support of political representatives | 5 |
| BLG11 | | Improve the inter-institutional communication by meeting for consensus and agreements | 5 |
| BSD6 | | Health professionals outreach activities ( going to houses and rural areas) | 4 |
| BSD10 | | Enhancement of laboratories | 4 |
| BHW9 | | Assist and monitor all diagnostic and treatment activities in the field | 4 |
| F3 | | Financing through credits by multilateral organizations | 4 |
| BSD1 | | Mechanisms and flowcharts of care for people detected in prevention and control activities | 3 |
| BSD11 | | Equipment improvement | 3 |
| BHW5 | | Training and supporting health workforce from relevant related programs | 3 |
| BHW6 | | Training in charge of Ministry of Health professionals and departmental Chagas representatives | 3 |
| BHW7 | | Training in charge of peers previously trained | 3 |
| BMVT5 | | Get Nifurtimox and Benznidazol registered in the national list of essential drugs | 3 |
| BLG15 | | Chagas law | 3 |
| BSD9 | | Mobile cardiograms | 2 |
| BHW1 | | Additional Human Resources | 2 |
| BHW10 | | On the job training (internships...) | 2 |
| BLG3 | | Assistance to scientific events, local, regional and international | 2 |
| BLG7 | | Publication of articles, manuals and other informative materials for expanding and socializing the model | 2 |
| BLG13 | | Activities for the validation of a new simplified diagnostic protocol | 2 |
| BSD5 | | Performing regular external quality control on laboratories | 1 |
| BMVT4 | | Reference to organizations offering pacemakers | 1 |
| F2 | | Financing through specific grants by cooperation mechanisms | 1 |
| F4 | | Implementing a contribution system | 0 |
