# Supplementary material for: Multi-criteria decision analysis approach for strategy scale-up with application to Chagas disease management in Bolivia
Source: PLoS Negl Trop Dis. 2021 Mar 26;15(3):e0009249. doi: 10.1371/journal.pntd.0009249 (PMC8026069; doi:10.1371/journal.pntd.0009249)
Supplement: S1 Text — (DOCX) [file pntd.0009249.s004.docx]

**S1_Text_Survey to experts in order to establish relative weight of each intervention.**

1. In relation to its impact, this intervention belongs to

- Effectiveness at the individual level

- Effectiveness at a population level

- Patient reported health status

- Security

- Equitable health distribution

- Perceived quality of patient care

- Reduced burden of disease

- Catastrophic Health Care Spending

- Economic productivity and care for others

2. Select the target group for this intervention

- Population with the infection

- Population at risk of acquire infection

- Total population of intervention area

3. Within its target group (population with the infection, population at risk or total population) it chooses the percentage directly affected by this intervention:

- <25% (0,25)

- 25-50% (0.5)

- 50-75% (0,75)

- >75% (1)

4. Can this intervention be carried out with the capacities of the service offered by the health system?

- Yes

- No

5. Select the necessary or most appropriate service requirements for implementation (if you answered yes to the previous question, skip to question number 6)

- Mechanisms and flow of healthcare for patients diagnosed in prevention and control activities

- Mechanisms for referring patients to the corresponding specialties

- Telephone network for patient care

- Readjustment of the hospital infrastructure

- Periodic external quality controls of the laboratories

- Phone calls to search for patients who do not come to the healthcare facility

- Providing diagnostic tools (such as EKG-Holter) at the primary and secondary healthcare level

- Use of mobile EKGs

- Strengthening of laboratories

- Strengthening of the equipment

- Provision of resources for blood banks and transplant units

- Management and organization of first and second level health facilities for standardized Chagas healthcare

6. Can this intervention be carried out with the Human Resources capacities offered by the health system?

- Yes

- No

7. Select the human resource requirements needed or most appropriate for implementation (if you answered yes to the previous question, skip to question number 8)

- Additional Human Resources

- Reinforce theoretical and practical knowledge about Chagas disease in all human healthcare professionals

- Training for specialists in charge of the healthcare of Chagas disease

- Training to the primary healthcare personnel

- Training of relevant specific program (Chagas program) staff

- Necessary training by professionals

- Training possible by previously trained personnel (train of trainer’s strategy)

- Training based of volunteers already trained and community leaders

- Assistance and monitoring of diagnosis and treatment in primary healthcare

- Training through clinical practices (exchanges, internships...)

- 100% practical (in healthcare centers) staff training

- 100% online staff training

- Combination of practical/ online training

- Revision, updating and dissemination of guidelines and manuals

- Allocation of more teaching hours for the diagnosis and treatment of Chagas within the medical curricula

- Incentive system for health professionals in relation to Chagas

- Individualized analysis together with medical staff in charge of a specific area based on activity reports (to analyze its congruence with the prevalence of the disease in the area)

8. Is this intervention feasible with the necessary Information Systems?

- Yes

- No

9. Select the actions you consider necessary to implement this intervention (if you answered yes to the previous question, skip to question number 10)

- Monitoring and quality control of the information generated by healthcare facilities

- Committee for the analysis of indications

- Integrated software for the management and analysis of the information generated in the health centers

10. Is this intervention easy to implement in terms of current accessibility to needed medicines and medical products?

- Yes

- No

11. Select the actions you consider necessary to implement this intervention (if you answered yes to the previous question, skip to question 12)

- Forecasting the demand for medicines

- External support to the supply chain of medicines and supplies from the national program (support networks in case of stock-outs, purchase circuit, etc.)

- Supply of supplies (pacemakers) and essential drugs for the treatment of Chagas disease by the Health System

- Register NFX and BNZ in National Regulatory Agencies

12. Estimated unit cost of the intervention per patient

- <5€ (0,25)

- >5 a ≤20€ (0,5)

- >20 a ≤50€ (0,75)

- >50€ (1)

13. Estimated impact on the public budget

- It does not impact the public budget or the expenditure is negligible (0)

- Slightly increases the budget (less than 1%) (0.25)

- Moderate increase in budget (between 1% and 5%) (0.5)

- Significantly increases the budget (between 5% and 10%) (0.75)

- Increases budget by more than 10% (1)

14. Is this intervention easy to maintain over time due to a sustainable source of funding?

- Yes

- No

15. Select the actions you consider necessary to implement this intervention (if you answered yes to the previous question, skip to question number 16)

- Financing through increased spending in the Chagas Health System

- Financing through specific collaboration mechanisms

- Financing through credits granted by multilateral organizations

- Implement a tax system (based on wage rates etc.)

16. Is this intervention easy to implement with current leadership and governance capabilities?

- Yes

- No

17. Select the actions needed to implement this intervention (if you answered yes to the previous question, continue on the next page)

- Development of an intervention plan

- Introducing Chagas into health system planning

- Attendance at local, regional and international scientific events

- Chagas disease symposia and operational models to increase diagnosis and treatment

- Community leaders involvement

- Meetings and advocacy workshops with representatives of institutions and organizations of municipalities involved

- Publication of articles, manuals or other informative supports to disseminate and socialize the model

- Leadership and support of programs by political representatives

- Health policy in relation to Chagas disease at the national level

- To designate coordinators in the programs of control of the Chagas disease in the different health areas

- Improve inter-institutional communication through meetings for agreements and joint action

- Participate in technical discussion tables to support the development / updating of the national protocol for diagnosis and treatment

- Joint action agreements with health institutions and services in the health network

- Chagas' Law and official regulations
