# Supplementary material for: Multi-criteria decision analysis approach for strategy scale-up with application to Chagas disease management in Bolivia
Source: PLoS Negl Trop Dis. 2021 Mar 26;15(3):e0009249. doi: 10.1371/journal.pntd.0009249 (PMC8026069; doi:10.1371/journal.pntd.0009249)
Supplement: S2 Text — (DOCX) [file pntd.0009249.s005.docx]

**S2_Text_Mathematical details to build the model**

The software used to develop a Multi Criteria Decision Analysis model was R (version 2.11.1) and R studio (version 1.0), combining and adapting the standard AHP, MDCA and DEA models to our problem structure.

In a standard MDCA model there are two main elements: the options or Interventions to be evaluated and the multiple criteria used for that aim. In our model there is an extra element, the Actions supporting the health system’s building blocks.

Each Intervention I_j_ results in outcomes in the four outcome categories; however, each Intervention also has associated with it a complexity metric for each of the six building blocks. The complexity metric measures the difficulty in the scale-up of the Intervention to produce the expected outcomes. Our model adds to the literature in that we identify a set of Actions that can be taken to reduce the complexity in each Building Block for the set of chosen Interventions. In order to accomplish this, we included a new level in the complexity side of our performance matrix adding all the possible Actions required by the selected Interventions to address the complexity of the Interventions and improve their scale-up effectiveness. Therefore, we needed to compute another level of weights for each possible Action.

We used AHP in order to compute the relative weights for the decision criteria of the model using a pairwise comparison matrix obtained through surveys of a select group of decision makers (Suppl 1). Relative weights for the “complexity” criteria identified in the six “Building Blocks” set of the model were determined in the same survey evaluating their complexity on a scale from one to four.

Once the criteria weights were established, each potential “Intervention” was evaluated by its impact on the criteria in the “outcomes” group, and necessity of “Actions” on each of the “Building Blocks”. It is noted that the Building Blocks are needed to enable the outcomes associated with an Intervention- if more Actions from the building blocks are needed this increases the “complexity” associated with the success of an Intervention. For each supporting “Action” we determined its contribution to addressing the complexity of every Building Block criterion. The maximum potential for addressing each building block criterion’s complexity was determined by summing up the contributions of all the Actions supporting that criterion. Dividing the actual contribution towards a criterion by the maximum potential for that criterion yielded a relative effectiveness factor for each of the Actions. For instance, if A_1_, ..., A_r_ are Actions related to the criteria C_1_ the contribution potential or weight for Action Aj, represented as w_Aj_ is computed as follows:

Once we have one specific weight for each of the columns of the performance matrix the computation of the Outcomes and Complexity scores is automatically performed by the tool, through the standard MCDA performance matrix.

We represented the group of n Interventions and k Actions as I = {I_1_, ..., I_n_} and Ac = {Ac_1_, ..., Ac_k_} respectively.

We define a Strategy (S) as a set containing a subset of the possible Interventions (I) and Actions (A), i.e. S = {S_I_, S_A_ |S_I_ ⊆ I, S_A_ ⊆ Ac)}.
